# Prevalence and clustering of cardiovascular risk factors among resident of coastal areas in Qinzhou, Guangxi, China

**DOI:** 10.1186/s13019-023-02137-0

**Published:** 2023-02-10

**Authors:** Fang He, Zhennan Liao, Yu-Mei Li, Yuanling Luo, Lili Wu, Liping Lin, Ying Chen, Weihong Deng, Junzhang Huang

**Affiliations:** Department of Nursing, The Second People’s Hospital of Qinzhou, No.219 of Wenfeng South Road, Qinzhou, 535000 China

**Keywords:** Cardiovascular diseases, Risk factor, Nationality

## Abstract

**Objective:**

We aimed to estimate the prevalence of CRFs and investigate its associated social-economic factors among adults in coastal areas of Qinzhou, Guangxi.

**Methods:**

A representative sample of 1836 participants aged 20 to 70 years was included in Qinzhou, Guangxi in 2020. Data were collected by the questionnaire, anthropometric and laboratory measurements. The prevalence of CRFs, including hypertension, dyslipidemia, diabetes, overweight or obesity, alcohol consumption, and smoking were calculated by standardization. The multivariate logistic regression analysis was performed to explore the independent factors associated with the presence of CRFs.

**Results:**

The age-standardized prevalence of hypertension, dyslipidemia, diabetes, overweight or obesity alcohol consumption, and smoking was 42.7%, 39.5%, 0.9%, 38.5%, 18.4% and 15.7%, respectively. The prevalence of clustering of at least one and at least two cardiovascular disease risk factors were 82.2% and 45.3% in total. There were differences in the aggregation of cardiovascular risk factors among different age, education, and income levels. There appeared higher clustering of at least one and at least two CRFs among adults with lower education level, higher income level and those elderly.

**Conclusions:**

Compared with other regions in China, a higher prevalence of CRFs exists among adults in Guangxi and several social-economic factors were associated with the presence of CRFs. These findings suggest that we should implement effective measures to control the CRFs, to reduce the risk of cardiovascular disease in adults.

## Introduction

The burden of cardiovascular disease (CVD) is increasing in China, at present, there are about 290 million individuals suffered from CVD. In 2016, the CVD mortality rate of rural resident of coastal areas and urban resident of coastal areas was 309.33/100,000, 265.11/100,000, respectively. Recently, cardiovascular disease is already the main cause of death in China, two out of every five deaths are due to cardiovascular disease [1, 2]. It has reported that hypertension, dyslipidemia, diabetes, overweight or obesity and alcohol consumption are five major cardiovascular risk factors (CRFs) [3, 4]. The recent study detected hypertension in 23.9%, dyslipidemia in 28.9%, diabetes in 6.2%, overweight or obesity in 35.6%, and alcohol consumption in 25.4% in the healthy adults of China from the 2011 Nanjing Chronic Disease and Risk Factor Surveillance [5, 6]. Clustering of two or more CRFs are common in the crowd [7–9], for example, 60% of patients with hypertension have a diagnosis of diabetes, and 74% of dyslipidemia [10]. There is a significant increase of the risk of CVD events with the accumulation of the CRFs [11]. Numerous studies have found the clusters of CRFs in China, such as the prevalence of CRFs and their clustering by Yu et al. [12] and Xu et al. [13], these studies demonstrated that the clustering of CRFs have widespread in China. However, most studies of CRFs were conducted in Han resident of coastal areas [14–16], the clusters of CRFs in inhabitant in Guangxi, China remain uncertain.

Therefore, we aimed to investigate the prevalence of CRFs in Guangxi with a focus on their clustering in population. Furthermore, we also explored the potential variables associated with the presence of CRFs in healthy residents of the region.

## Methods

### Study population

The participants were recruited from Qinzhou, Guangxi in 2020, according to the population size and mobility of the project site, the convenience sampling method is adopted, with a total sample size of 1836 without a history of CVD. There are 56 nationalities in China and Zhuang ethic group is the largest minority. Qinzhou is a coastal area of Guangxi Beibu Gulf, which has two urban areas, two counties and one national development zone, including 54 towns and 12 streets. Most of the population in this area go out to work, and the proportion of women who stay behind is higher. However, they are all local residents and can better reflect the local epidemiological situation. Considering the geographical characteristics of coastal areas, one national development zone and 15 coastal towns were included in the sampling process. Cluster sampling method was used to extract people aged 20 to 70 years old and a total of 1831 resident of coastal areas participated in the study, excluding 55 participants with missing data. Rural residents accounted for 843 cases (45.89%), and urban residents accounted for 994 cases (54.11%). All participants signed a written informed consent from during recruitment.

### Data collection

We collected all data by direct interviews. Using a standard questionnaire to survey participants, including gender, age, residence, education (elementary school and below, junior high school or high school, college and above) and annual family income (< 10 thousand RMB per year, 10 ~ 30 thousand RMB per year, > 30 thousand RMB per year); life style, including and alcohol consumption (no, yes); and self-reported previous medical history.

Physical measurements included weight, height and blood pressure (BP). Measuring the weight to the nearest 0.1 kg and height was measured the nearest 0.1 cm. Weight was divided by square of height (in meters) to calculate Body Mass Index (BMI) of participants. Before BP measurements, participants were advised to avoid engaging in physical activities. BP was measured after at least 5 min of rest.

Blood samples were collected after fasting for at least 8 h. Individuals were test an OGTT with 75 g glucose after collecting the blood samples of fasting plasma glucose (FPG)and serum lipids. Biochemical indicator measurements included plasma glucose and four items of blood lipids (total cholesterol (TC), triglycerides (TG), low-density lipoprotein cholesterol (LDL-C) and high-density lipoprotein cholesterol (HDL-C).

### Assessment criteria

The five major CRFs were defined as follows: according to standard criteria in Chinese, hypertension was defined as an average systolic blood pressure (SBP) ≥ 140 mmHg and/or an average diastolic blood pressure (DBP) ≥ 90 mmHg and/or reported a history of hypertension or taking antihypertensive drugs [17].Dyslipidemia was defined as presence of at least one of the following: TG ≥ 2.30 mmol/L, TC ≥ 6.20 mmol/L, HDL-C < 1.0 mmol/L and LDL-C ≥ 4.1 mmol/L, and/or use of antilipidemic drug [18]. And diabetes was defined as FPG ≥ 7.0 mmol/L, or OGTT ≥ 11.1 mmol/L, or a self-reported history of diabetes [19]. Overweight and obesity were defined as BMI of 24.0–27.9 kg/m^2^ and ≥ 28.0 kg/m^2^, respectively [20]. Alcohol consumption defined as drinking more than twice a month [21]. Having at least two CRFs in one individual CVD was defined risk factor clustering [12]. The prevalence rate, also known as the prevalence rate, refers to the proportion of new and old cases of a disease in the total population at a specific time. Prevalence = (number of new cases of a certain disease in a certain population in a certain period/number of exposed population in the same period) × 100%.

### Statistical analysis

All statistical analyses were performed using SPSS 19.0 (IBM Corp. Silicon Valley, CA, US). Quantitative data were expressed as mean ± standard deviation. Categorical data were expressed as percentage while Chi-square test and Fisher’s exact test were using in intergroup comparing. And the CRFs clusters were analyzed through multi-classification logistic regression. The prevalence of CRFs were standardized to the age of the population according to the Chinese sixth national population census. The multivariate logistic regression analysis was performed to examine whether age, gender, education level and family income level were associated with the presence of CRFs corrected by age and gender. A *P* value of < 0.05 were considered statistically significant.

The calculation of sample size was performed using PASS software version 15.0.5 (NCSS, LLC. Kaysville, Utah, USA). According to the previous literatures [1–3], we assumed that there was an average prevalence about 30% for cardiovascular risk factors in Qinzhou region. We tried to select a tolerance error of 0.1 times the estimated population proportion, which in this case was 3%, and a confidence (1-alpha) of 0.95 was set. The exact (Clopper-Pearson) formula was used to calculate the confidence interval for a proportion in this cross-sectional study and a minimum sample size of 928 is recommended. In addition, we assumed that the non-response rate of the study subjects is 10% and the qualified rate of the questionnaire is 10%, so the sample size required for this study is 1146 cases.

## Results

### Descriptive characteristics of participants

There were 1836 subjects (20–70 years, mean age 52.56 ± 11.72 years), including 680 (37.0%) men and 1156 (63.0%) women; 30.4% of participants were aged 45–54 years, and 30.2% of participants were aged 55–64 years. There were 529 (28.8%) individuals having an education level of primary school and lower, 1131 (61.6%) having junior or senior high school, and 176 (9.5%) having college and higher. There were 578 (31.4%) participants with an annual income of < 10,000 yuan, 950 (51.7%) participants with 10,000–30,000 yuan, and 308 (16.7%) participants with > 30,000 yuan.

### Detection of cardiovascular risk factors

In this study, we found that the age-standardized prevalence of hypertension, dyslipidemia, diabetes, overweight or obesity alcohol consumption, and smoking was 42.7%, 39.5%, 0.9%, 38.5%, 18.4% and 15.7%, respectively. There was a statistically significant difference in the prevalence of hypertension, and overweight/obesity between the age group (*p* < 0.001 for all). The prevalence of hypertension and overweight or obesity and were highest in ≥ 65 years old. Men have a higher prevalence of drinking and smoking than women (*p* < 0.001). There were statistically significant differences in the rates of dyslipidemia and overweight/obesity among people with different income levels (*p* < 0.05). There was a negative trend in the original and standardized prevalence of hypertension, dyslipidemia, and diabetes mellitus by level of education (Table 1).

**Table 1:** The prevalence of related cardiovascular risk factors

Category	Number	Hypertension, %	Dyslipidemia, %	Diabetes mellitus, %	Overweight/Obesity, %	Alcohol consumption, %	Smoking, %
Total^b^	1836	53.3 (42.7)	41.7 (39.5)	1.4 (0.9)	44.6 (38.5)	18.7 (18.4)	14.6(15.7)
Age, years^a^							
< 35	150	22.0	35.3	0	26.0	17.3	18.0
35–44	262	46.9	40.1	0.8	40.5	19.8	15.6
45–54	559	52.8	43.1	1.4	48.3	17.9	11.1
55–64	555	57.8	43.2	2.0	43.6	19.5	16.8
≥ 65	310	66.5	41.0	1.6	52.3	18.7	14.5
*p* value		< 0.001	0.426	0.365	< 0.001	0.930	0.056
Gender^b^							
Male	680	54.0 (42.4)	40.3 (40.0)	1.3 (0.7)	44.9 (40.2)	32.2 (31.3)	25.1(25.5)
Female	1156	52.9 (42.9)	42.6 (39.0)	1.5 (0.9)	44.5 (37.3)	10.8 (10.7)	8.4(9.7)
*p* value		0.644	0.342	0.797	0.871	< 0.001	< 0.001
Education^b^							
Primary school and lower	529	55.6 (48.7)	38.2 (40.4)	1.5 (1.0)	40.8 (41.7)	16.6 (20.6)	11.7(16.0)
Junior or senior high school	1131	54.0 (43.8)	43.1 (40.4)	1.5 (0.8)	46.9 (42.8)	20.8 (20.6)	16.5(16.7)
College and higher	176	41.5 (42.0)	43.2 (43.3)	0.6 (0.9)	40.9 (40.2)	11.9 (11.6)	10.8(11.1)
*p* value		0.004	0.148	0.606	0.038	0.007	0.011
Income, thousand yuan per year^b^							
< *10*	578	55.2 (45.1)	38.9 (35.8)	1.0 (0.6)	41.3 (37.2)	19.0 (16.7)	15.7(17.1)
* 10* ~ *30*	950	51.9 (41.1)	40.9 (39.6)	1.8 (1.1)	43.2 (35.3)	19.8 (21.9)	14.4(16.7)
> *30*	308	53.9 (43.5)	49.4 (45.6)	1.0 (0.8)	55.2 (49.6)	14.9 (12.0)	13.0(11.4)
*p* value		0.443	0.009	0.373	< 0.001	0.161	0.529

^a^The data in the table is the original prevalence

^b^The data in the parentheses is the age-standardized prevalence and the data outside the brackets is the original prevalence

The statistical analyses in the table use the original prevalence

### The standardized prevalence of CRFs by gender

There were significant differences in the prevalence of hypertension in all age groups (*p* < 0.001). The differences in overweight/obesity rates and smoking rates among different age groups were statistically significant in women (*p* < 0.001) was not statistically significant in the male group. There were differences in the prevalence of hypertension, overweight/obesity and alcohol consumption among men at different educational levels (*p* < 0.05), and there was a trend of increasing with the increase of education level. Overweight/obesity rates varied across income levels for both men and women (*p* < 0.05), there were differences in the rate of dyslipidemia among women with different income levels (*p* < 0.05), there were differences in alcohol consumption rates among men with different income levels (*p* < 0.05) (Table 2).

**Table 2:** The prevalence of major cardiovascular disease (CVD) risk factors by relevant characteristics stratified by gender

Category	Hypertension, %		Diabetes, %		Dyslipidemia, %		Overweight or obesity, %		Alcohol consumption, %		Smoking, %	
	Men	Women	Men	Women	Men	Women	Men	Women	Men	Women	Men	Women
Age group, years^a^												
< 35	20.6	23.0	0.0	0.0	39.7	32.2	30.2	23.0	28.6	9.2	27.0	11.5
35–44	45.2	47.8	0.0	1.1	38.1	41.0	42.9	39.3	33.3	13.5	25.0	11.2
45–54	53.8	52.2	1.0	1.7	44.2	42.5	47.7	48.6	31.7	10.3	22.1	5.0
55–64	58.0	57.8	1.9	2.0	37.7	46.6	44.9	42.8	33.3	11.2	27.5	10.3
≥ 65	70.1	63.9	2.4	1.1	40.2	41.5	48.8	54.6	32.3	9.3	25.2	7.1
*p* value	< 0.001	< 0.001	0.439	0.658	0.728	0.174	0.130	< 0.001	0.965	0.707	0.788	0.033
education ^b^												
Primary school and lower	54.4 (55.9)	56.1 (55.0)	0.6 (11.9)	2.0 (9.0)	39.8 (48.8)	37.4 (54.2)	41.5 (36.4)	40.5 (42.1)	32.7 (58.3)	8.9 (4.0)	23.4	6.1
Junior or senior high school	56.9 (52.1)	52.2 (49.3)	1.9 (7.0)	1.3 (8.9)	40.0 (47.3)	45.1 (50.5)	48.6 (42.5)	45.9 (46.1)	35.2 (54.9)	11.9 (2.4)	27.5	9.7
College and higher	36.4 (39.7)	45.5 (33.1)	0 (5.2)	1.0 (8.9)	42.9 (43.1)	43.4 (42.7)	31.2 (46.9)	48.5 (40.9)	14.3 (50.2)	10.1 (2.7)	15.6	7.1
*p* value	0.004	0.146	0.263	0.642	0.886	0.058	0.011	0.172	0.001	0.337	0.069	0.122
Income, thousand yuan per year^b^												
< 10	56.5 (43.0)	54.4 (46.0)	0.9 (0.4)	1.1 (0.7)	40.8 (38.3)	37.7 (34.4)	39.9 (33.5)	42.3 (39.2)	28.7 (21.9)	13.0 (13.1)	25.1 (21.6)	9.9 (13.8)
10 ~ 30	52.5 (41.6)	51.5 (41.1)	1.9 (1.0)	1.7 (1.0)	38.6 (39.5)	42.4 (39.6)	44.2 (38.8)	42.5 (32.5)	36.4 (38.9)	9.7 (10.3)	26.1 (28.9)	7.3 (7.8)
> 30	53.6 (42.4)	54.0 (42.6)	0.0 (0.0)	1.4 (1.1)	45.4 (45.2)	51.2 (45.2)	58.8 (54.5)	53.6 (45.6)	24.7 (19.1)	10.4 (7.8)	21.6 (18.9)	9.0 (7.1)
*p* value	0.640	0.650	0.263	0.780	0.476	0.007	0.007	0.013	0.037	0.281	0.668	0.362

^a^The data in the table is the original prevalence

^b^The data in the parentheses is the age-standardized prevalence and the data outside the brackets is the original prevalence

### Prevalence in clustering of CRFs

Overall, 17.9%, 36.9% and 45.3% of participants had zero, one and at least two CRFs. The prevalence of zero, one and at least two CRFs were 24.3%, 38.8%, and 36.9% in male, 27.6%, 36.3%, and 45.6% in female, respectively. There were differences in the aggregation of cardiovascular risk factors among different age, education, and income levels (Table 3).

**Table 3:** The prevalence with different numbers of cardiovascular disease (CVD) risk factors

Category	None (0)	Single (1)	Clustering (> = 2)	*P* value
Total, n (%)	17.9 (26.3)	36.9 (36.4)	45.3 (37.3)	
Gender ^b^				0.818
Male	17.5 (24.3)	37.8 (38.8)	44.7 (36.9)	
Female	18.1 (27.6)	36.3 (35.1)	45.6 (37.3)	
Age group^a^				< 0.001
< 35	44.0	33.3	22.7	
35–44	21.0	41.2	37.8	
45–54	16.1	36.3	47.6	
55–64	15.7	37.7	46.7	
≥ 65	9.7	34.5	55.8	
Education ^b^				0.002
Primary school and lower	19.5 (21.9)	37.2 (36.7)	43.3 (41.4)	
Junior or senior high school	15.6 (22.7	37.7 (38.1)	46.8 (39.2)	
College and higher	27.8 (28.5)	30.7 (29.7)	41.5 (41.8)	
Income, thousand yuan per year ^b^				0.011
< 10	18.7 (28.3)	38.4 (35.9)	42.9 (35.8)	
10 ~ 30	18.4 (26.2)	37.9 (38.6)	43.7 (34.8)	
> 30	14.6 (22.3)	30.8 (30.9)	54.5 (46.8)	

^a^The data in the table is the original prevalence

^b^The data in the parentheses is the age-standardized prevalence and the date outside the brackets is the original prevalence

### Multivariate logistic regression analysis for cardiovascular disease risk factors

As shown in Table 4, there is no difference in the risk of only one CRF among all groups. Those with an annual income of more than 30,000 yuan have a higher risk of having two OR more CRFs (OR: 1.536) than those with an annual income of less than 10,000 yuan. Those with a college education OR higher had a lower risk of having 2 or more CRFs than those with a primary school education or less (OR: 0.001). There was an interaction between age and education level on CRF aggregation. Compared with those aged < 35 and those with primary school education OR below, those with college education or above and those aged 35–44, 45–54, 55–64 and 65 and above had a higher risk of two or more CRFs (OR: 22.306, 11.737, 12.496 and 14.753, respectively).

**Table 4:** The multivariate logistic analysis of the cardiovascular disease (CVD) risk factor clustering

	Single (1)			Clustering (≥ 2)		
Category	OR	95%CI	*P* value	OR	95%CI	*P* value
Gender:						
Female	1.000					
Male	1.073	0.88 to 1.309	0.485	0.971	0.797 to 1.183	0.770
Age						
< 35	1.000					
35–44	1.232	0.463 to 3.275	0.676	1.037	0.397 to 2.706	0.941
45–54	1.032	0.413 to 2.584	0.946	1.131	0.462 to 2.767	0.787
55–64	1.444	0.577 to 3.614	0.432	0.918	0.373 to 2.26	0.852
≥ 65	0.886	0.346 to 2.273	0.802	1.788	0.718 to 4.457	0.212
Income, thousand yuan per year						
< 10	1.000					
10 ~ 30	0.983	0.793 to 1.22	0.879	1.029	0.83 to 1.276	0.793
> 30	0.753	0.551 to 1.028	0.074	1.536	1.134 to 2.08	0.006
Education:						
Primary school and lower	1.000					
Junior or senior high	1.073	0.397 to 2.903	0.889	0.605	0.225 to 1.624	0.318
College and higher	0.866	0.311 to 2.416	0.784	0.108	0.029 to 0.404	0.001
Education Age						
Primary school and lower by < 35 years old	1.000					
Junior or senior higher by 35-44 years old	1.228	0.392 to 3.846	0.724	1.073	0.343 to 3.357	0.903
Junior or senior high by 45-54 years old	1.009	0.347 to 2.928	0.987	2.097	0.731 to 6.019	0.169
Junior or senior high by 55-64 years old	0.697	0.240 to 2.024	0.507	2.686	0.930 to 7.753	0.068
Junior or senior high by ≥ 65 years old	1.213	0.400 to 3.679	0.733	1.713	0.574 to 5.109	0.334
College and higher by 35-44 years old	0.572	0.134 to 2.439	0.451	22.306	4.448 to 111.852	< 0.001
College and higher by 45-54 years old	1.516	0.426 to 5.393	0.520	11.737	2.601 to 52.957	0.001
College and higher by 55-64 years old	0.827	0.226 to 3.024	0.774	12.496	2.735 to 57.094	0.001
College and higher by ≥ 65 years old	0.953	0.213 to 4.256	0.949	14.753	2.768 to 78.647	0.002

The multivariate logistic regression analysis was performed to examine whether age, gender, education level, and family income level were associated with the presence of CRFs corrected by age and gender

## Discussion

In this study, we explored the prevalence of CRFs in healthy adults in the region of Qinzhou and evaluated the association between several social-economic factors and the presence of CRFs. The main findings can be summarized as follows: (1) there was a relatively high prevalence of CRFs in the healthy population in Qinzhou; (2) several factors such as age, income and education were associated with the presence of CRFs. To the best of our knowledge, this is the latest study to report the prevalence and clustering of major CRFs in resident of coastal areas. Our results revealed the high prevalence of CRFs in this region and emphasized the importance of early prevention of adverse cardiovascular events. More attention should be paid to the control of these social-economic factors to achieve the precision prevention.

Guangxi is a concentrated region of ethnic minorities, is the most populous minority in Guangxi. Resident of coastal areas have unique lifestyle characterized by rice-based food, high intake of salt and oil, whereas low intake of milk, eggs and fruits. Furthermore, resident of coastal areas like to drink alcohol [21, 22].

This study demonstrated that the age-standardized prevalence of hypertension, dyslipidemia, diabetes, overweight or obesity alcohol consumption, and smoking was 42.7%, 39.5%, 0.9%, 38.5%, 18.4% and 15.7%, respectively. The prevalence of hypertension, dyslipidemia, and diabetes was lower than a previous study among Tibetan in China (62.4%, 42.7%, and 6.4%), whereas the prevalence of overweight or obesity were higher than Tibetan adults (34.3%) [13]. The prevalence of the CRFs increased with age except of alcohol consumption. Higher prevalence of alcohol consumption present in men than in women, even the proportion of man was much smaller than woman. This phenomenon may result from the fact that men are more likely to drink alcohol than women under the influence from local culture and customs. The high prevalence of hypertension and dyslipidemia in adults in this study may due to the diet habits such as the high intake of salt and oil, which were the well-known risk factors of hypertension and dyslipidemia [23]. The location of this survey was selected in several local township, because there were more migrant workers, the left-behind population was insufficient, so the number of investigations was small. But all the respondents were local residents, this study was still representative. Because migrant workers are mostly men, this also explains why the proportion of women is higher than men. [24].

A recent study confirmed that the prevalence of hypertension (54.6%), overweight or obesity (24.5%) and smoking (35.8%) among Kazakh was higher than other ethnic groups in China, dyslipidemia prevalence (54.3%) was higher among Uygur, and diabetes prevalence (7.1%) was higher among Hans [25]. In the present study, the prevalence of the hypertension, diabetes, dyslipidemia, overweight, and current smoking were 24.3%, 4.3%, 49.3%, 32.0%, and 21.7%, respectively [26]. Another study in rural Nepalese aged 40–80 years reported that the prevalence of current smoking, Overweight and obesity, hypertension, diabetes and dyslipidemia were 24.1%,59.4%, 42.9%,16.2% and 56.0%, respectively [27].

Numerous studies have indicated that CVD incidence related to the clustering of CVD risk factors [28, 29]. Previous studies confirmed that the clustering of CRFs has a more harmful cardiovascular effect [30–33].

In this study, we found that 82.2% and 45.3% of participants had one or more and two or more CRFs. In a representative sample of 23,010 adults from the 2007–2011 cross-sectional survey, the proportions of respondents had one or more and two or more CRFs were 70.3% and 40.3%, respectively [26]. Compared with this study, Guangxi inhabitants had a higher proportion of at least one CRFs. In another cross-sectional survey of 16,371 Chinese suburban resident of coastal areas aged 35 to 74 years, 83.5%, 47.2% of people had at least one and at least two CRFs, respectively [34]. Compared this study, Guangxi resident of coastal areas had a lower prevalence of at least two CRFs. Other countries have also observed the clustering of CRFs. In the United States, most participants (80% of men, 71% of women) had at least one risk factor [35]. Clustering of at least three CRFs was observed in 69.4% of the men and 58.5% of the women in rural Nepalese [27]. In Malaysia, one third of the participants had at least two CRFs [36]. Our study demonstrated that the elderly, and the participants having lower levels of education were more likely to have one or more CRFs compared with the young and had higher levels of education.

Our research has several limitations. First at all, there exist large imbalance between genders, which may give major weight to female populations and influence the results in multinomial logistic analysis. The OR value in single CRF might be overestimate. Second, the sample size was not large enough in this study, the sample size will be expanded for further investigation in follow-up studies. In the future, subsequent large, multicenter studies are warranted to well report the prevalence of CRFs in different subgroups in real-world situation and investigate the causal relationship between social-economic factors and the presence of CRFs in a long period of follow-up. More effort should be undertaken on this topic to improve the primary prevention of adverse cardiovascular events.

## Conclusion

In conclusion, there was a relatively higher prevalence of CRFs among the healthy adults in the Qinzhou region compared with other regions and several social-economic factors were associated with the presence of CRFs. More effort should be taken to control these social factors and prevent the occurrence of CRFs, thus reducing the risk of cardiovascular disease in healthy people.

## Data Availability

All data generated or analyzed during this study are included in this article.
